# The Ear and Aviation

**Published:** 1918

**Authors:** 


***The Ear and Aviation.*** By Isaac H. Jones, Major M. R. C. Abs. from *Jour. A. M. A.*, Nov. 10, 1917.

In order to preserve the wonderful accuracy necessary in controlling such a delicate mechanism as the flying machine, the aviator relies preeminently on his *ear* balance-sense. It is highly probable that many an aviator has gone to his death because, all unknown to him, he did not possess a normal ear mechanism. Even when an individual is standing or walking on the earth, his ears constitute his sense organs of balance; and he has in addition the contributory help of information received from his muscle sense and his sight. When he rises above the earth and flies in the dark it is obvious that these contributory factors are practically eliminated and that he must rely almost exclusively on the ear-balance sense.

The following briefly summarizes the fundamental principles underlying the examination for aviators. The aviator must have 20/20 vision without glasses. He must have 40/40 hearing. He must be a normal man, with the additional requirements of a certain relative height, weight, and chest measurement, and a definite vision and auditory acuity. Moreover, he must have a good *balance-mechanism*.

The tests for nystagmus, pointing and falling[1] are merely to determine whether or not the ear mechanism is normal. A limited number of candidates give what might be termed " border line " responses. It is here that the caloric test is useful. Water at 68° F. is allowed to run into the external auditory canal from a height of about 3 feet through a stop nozzle, with the head tilted 30 degrees forward, until the eyes are seen to jerk or the individual becomes dizzy. The length of time shown by the stop-watch in the normal

1. Some Observations on the Barany Tests as Applied to Aviators, p. 563.

is 40 seconds. The type of nystagmus is then noted; it should be rotary and the direction of the jerk should be to the side opposite the ear douched. The eyes are then closed and the past-pointing taken. The head is then immediately inclined backward 60 degrees from the perpendicular (or 90 degrees from the original position), when there should appear a *horizontal* nystagmus to the side opposite the ear douched. The eyes are now closed, and the past-pointing taken with the head in this position; after which the left ear is douched and the same procedure carried out. If the caloric test applied in one of these " border line " cases shows only a slight impairment of the responses from each ear, the candidate is qualified. A slight impairment would be indicated if, nstead of the normal forty seconds of douching, there was required not more than ninety seconds. If one ear shows normal responses, whereas the other shows responses only after *more* than ninety seconds of douching, the candidate is disqualified.

Many candidates feel that " jumping eyes " and evidences of vertigo are signs of weakness and would be counted against them. Supposing that the candidate, after having been urged " perfectly naturally, " still fails to past-point, although he has shown a normal nystagmus and falling, the quantitative estimation of vertigo is taken. Should the candidate show over sixteen seconds of vertigo in both directions, the examiner then realizes that the absence of past-pointing was probably due to a calculated correction. The question is definitely settled by douching the ears. Although a candidate can estimate the significance of the sensation of vertigo after turning, he has no control over this sensation after douching; he is unable to calculate the meaning of the vertigo produced by the caloric test. Therefore, if he fails to past-point after the douching tests, he is definitely disqualified.

